# Integrative biology shows DPP4 affects inflammatory response to eclampsia and cell model growth via p65/NLRP3/ASC/Caspase-1 pathway

**DOI:** 10.3389/fgene.2026.1775026

**Published:** 2026-03-04

**Authors:** Zhimin Bian, Li Hao, Rongjuan Yang, Jianghui Sun

**Affiliations:** Department of Obstetrics, Shijiazhuang Obstetrics and Gynecology Hospital, Shijiazhuang, Hebei, China

**Keywords:** DPP4, eclampsia, growth, inflammatory response, pathway

## Abstract

**Objective:** Eclampsia severely endangers maternal and neonatal health, being a major contributor to emergency admissions, maternal mortality, and long-term complications. This study aimed to identify reliable biomarkers and explore potential therapeutic targets for improving the diagnosis, prevention, and management of eclampsia.

**Methods:** Differential gene expression analysis was performed on the GSE60438 dataset. Weighted Gene Co-expression Network Analysis (WGCNA) was used to construct gene modules and screen modules associated with pre-eclampsia. Gene Ontology (GO) and Gene Set Enrichment Analysis (GSEA) were employed to annotate the biological functions and pathways of candidate genes. Immune cell infiltration was evaluated via the xCell algorithm. LASSO regression was utilized to identify hub genes, which was validated by RT-qPCR and Western blot in clinical samples (placental tissues and serum from pre-eclampsia patients). DPP4 knockdown experiments were conducted in HTR-8 cells to assess its effects on pro-inflammatory cytokines (IL-6, TNF-α) and trophoblast cell functions (migration, invasion, lumen formation). Additionally, the p65/NLRP3/ASC/Caspase-1 signaling pathway was examined to clarify the underlying molecular mechanism.

**Results:** A total of 4,642 upregulated and 2,193 downregulated genes were identified in pre-eclampsia samples. WGCNA revealed nine gene modules, with the red module showing the strongest positive correlation and the magenta module exhibiting a negative correlation with pre-eclampsia. GO analysis indicated enrichment of candidate genes in chromosome organization, mitochondrial function, and DNA repair. GSEA identified key immune-related pathways, including cytokine production and chemokine signaling. LASSO regression pinpointed DPP4 as a hub gene, which was significantly upregulated in pre-eclampsia clinical samples. DPP4 knockdown in HTR-8 cells reduced IL-6 and TNF-α levels, impaired trophoblast migration, invasion, and lumen formation, and inhibited the phosphorylation of p65, NLRP3, ASC, and Caspase-1 in the p65/NLRP3/ASC/Caspase-1 signaling pathway.

**Conclusion:** Targeting DPP4 may serve as an innovative strategy for regulating inflammatory signaling in eclampsia, with potential to alleviate maternal symptoms and improve pregnancy outcomes.

## 1 Introduction

Pregnancy-related disorders account for 1.3% of annual emergency room visits, and the incidence and mortality rates among women of reproductive age have increased significantly (Boushra et al., 2022; Hauspurg and Jeyabalan, 2022). Hypertensive disorders of pregnancy (HDP) are prevalent but non-linear conditions occurring during pregnancy and the postpartum period, contributing to nearly 15% of global maternal deaths (Rayes et al., 2023; Tschiderer et al., 2023). Among these, preeclampsia and eclampsia are the most severe forms of HDP, with eclampsia—though rare—responsible for the majority of these deaths (Ardissino et al., 2023; Tanner et al., 2022). Maternal mortality due to these conditions ranges from 5.6% to 14%, and approximately 25% of affected women experience long-term complications, including cardiovascular diseases such as myocardial infarction and heart failure (Goldstein and Pagidipati, 2022; Lindley and Davila-Roman, 2024). Newborns born to women with eclampsia are also at heightened risk for prematurity, hypoxic-ischemic brain injury, and neonatal respiratory distress syndrome (Minor et al., 2024). One study found that 38% of patients with acute eclampsia did not exhibit symptoms of preeclampsia during hospitalization (Rosenberg and Seely, 2024). Conversely, other studies have shown that, without intervention, preeclampsia and its features progress to eclampsia in 1.9% and 3.2% of cases, respectively (Erez et al., 2022). Therefore, identifying reliable biomarkers for eclampsia could greatly enhance its diagnosis, prevention, and management.

The pathogenesis of eclampsia is associated with an imbalance in angiogenesis, particularly involving the interplay between pro-angiogenic factors (such as VEGF, PlGF, and TGF-β) and anti-angiogenic factors (including sFlt-1 and sEng) (Hertig and Liere, 2010; Weel et al., 2016; Ives et al., 2020). Alterations in the concentrations of biomarkers like PlGF, sFlt-1, and sEng are closely linked to the onset of preeclampsia (Iannaccone et al., 2022; Foidart et al., 2009). Notably, during late pregnancy, the elevated levels of sFlt-1 and reduced levels of PlGF lead to endothelial dysfunction, contributing to the development of pathological manifestations such as hypertension, renal impairment, and proteinuria (Jardim et al., 2015). Monitoring the plasma concentrations of PlGF, sFlt-1, and sEng in combination enhances both the sensitivity and specificity of early eclampsia diagnosis. Moreover, the concentration changes of PAPP-A, a placental glycoprotein, in early pregnancy, have been identified as a potential biomarker for predicting eclampsia, particularly in cases of early-onset eclampsia. Other placental-secreted proteins, such as PP-13 (Spencer et al., 2007) and GDF-15 (Xu et al., 2024), have also been explored for early screening of eclampsia. By integrating these biomarkers with diagnostic tools like Doppler ultrasound, the accuracy of early eclampsia prediction can be significantly improved. However, the effectiveness of these methods remains debated, underscoring the need for continued exploration of novel biomarkers to enhance clinical management and prognostic accuracy.

The etiology of eclampsia is intricately linked to the dysregulation of inflammatory responses, with immune aberrations being a pivotal factor in its pathogenesis (Javandoust Gharehbagh et al., 2024). Despite ongoing research, the precise mechanisms and effective preventive and therapeutic strategies remain elusive. Studies have revealed a general downregulation of lymphocyte-mediated immune responses in preeclampsia patients, characterized by an increased proportion of regulatory T cells, a reduced count of natural killer cells, and a predominance of Th1-type immune responses (Kurmanova et al., 2024; Jianjun et al., 2010). Moreover, research has identified galectin-9 in early pregnancy maternal blood as a novel biomarker for the early prediction of eclampsia (Li et al., 2024). Placental-derived galectin-9 has been found to act as a regulatory molecule for a specific subpopulation of pro-inflammatory macrophages, inducing defects in uterine vascular remodeling and contributing to the development of preeclampsia (Li et al., 2018; Miko et al., 2013). Future research will continue to delve into the inflammatory mechanisms of eclampsia to develop more effective early predictive markers and therapeutic strategies.

In this investigation, we leveraged transcriptomic datasets from eclampsia cases to elucidate the potential pathogenic mechanisms underlying eclampsia progression through differential expression analysis and functional enrichment studies. Subsequently, employing Weighted Gene Co-expression Network Analysis (WGCNA) coupled with machine learning algorithms, we identified pivotal genes implicated in eclampsia development. To further substantiate our findings, we conducted validations using clinical specimens and cellular experiments, focusing on the biological role and underlying mechanisms of the key gene DPP4 in the context of eclampsia progression. Collectively, our research lays a robust theoretical groundwork for the exploration of biomarkers associated with eclampsia.

## 2 Materials and methods

### 2.1 Transcriptome data acquisition and analysis

The transcriptomic dataset GSE60438 (platform GPL6884) was retrieved from the Gene Expression Omnibus (GEO) database (Yong et al., 2015). This dataset was selected for its inclusion of clinically defined severe preeclampsia/eclampsia samples. For specific details, please refer to Supplementary Table S1. Probe IDs were mapped to official gene symbols using the ID Conversion tool available in SangerBox (http://sangerbox.com/). Differential expression analysis between the eclampsia patient group (N = 25) and the normotensive control group (N = 23) was performed using the limma R package. To control for false discoveries in this high-dimensional analysis, the resulting p-values were adjusted using the Benjamini-Hochberg false discovery rate (FDR) method. Genes meeting the thresholds of FDR-adjusted p-value <0.05 and an absolute log2 fold change (|log2FC|) > 1 were defined as significantly differentially expressed genes (DEGs). All data processing and statistical analyses were implemented in R, with data visualization conducted using the ggplot2 and pheatmap packages.

### 2.2 Weighted gene co-expression network analysis (WGCNA)

To identify hub genes associated with eclampsia pathogenesis, we performed WGCNA on the BioWinford platform (http://biowinford.site/). The gene expression data were normalized and filtered, focusing on the top 5000 most variable genes. A weighted neighbor-joining matrix was constructed based on gene correlations, and appropriate soft-thresholding was applied to ensure a scale-free network. Hierarchical clustering was used to divide the network into modules, which were then correlated with clinical features using Pearson’s coefficients. Hub genes were identified by high connectivity within modules, as determined by module membership (MM) and gene significance (GS). Co-expression networks were visualized using heatmaps and network diagrams to highlight key hub genes. Statistical analyses were performed in R, with P values <0.05 considered significant.

### 2.3 GO and GSEA functional enrichment analysis

To explore the biological functions and pathways associated with eclampsia, we conducted functional annotation and visualization using the BioWinford Platform. DEGs were subjected to Gene Ontology (GO) enrichment analysis, with a minimum of 3 overlapping genes and a p-value ≤0.01 considered statistically significant. Additionally, Gene Set Enrichment Analysis (GSEA) was performed to assess the differential expression of genes in eclampsia, focusing on GO terms. Ranked gene lists, based on logFC or t-values, were input into GSEA, with gene set sizes ranging from 15 to 500 and 1000 permutations. A significance threshold of p < 0.05 was applied.

### 2.4 Immune infiltration analysis

To assess immune infiltration in the control and eclamptic patient groups, we used the xCell algorithm on the BioWinford Platform, which provides detailed enrichment analysis of 64 immune and stromal cell types. The analysis was based on normalized gene expression data, and the results were visualized using box plots to compare immune infiltration levels. Statistical significance was assessed using the Wilcoxon rank-sum test, with p < 0.05 considered significant.

### 2.5 LASSO regression analysis

LASSO (Least Absolute Shrinkage and Selection Operator) regression was employed to refine the feature set derived from WGCNA, selecting the most parsimonious and discriminative genes associated with eclampsia. This analysis was conducted using the BioWinfordMR online platform (Wang et al., 2025). The optimal penalty parameter (λ) was determined through 10-fold cross-validation, which minimized the mean cross-validated error and thereby prevented model overfitting. This process resulted in a simplified model that retained only the most critical predictive features.

### 2.6 Sample collection and processing

Eclampsia patients and healthy controls were recruited from the Department of Obstetrics. Eclampsia was diagnosed according to international guidelines (ISSHP/ACOG): new-onset tonic-clonic seizures in a woman with preeclampsia (hypertension ± proteinuria/organ dysfunction after 20 weeks of gestation), after excluding other causes. Exclusion of alternative seizure etiologies (e.g., epilepsy, stroke, metabolic disorders) was performed through detailed history, neurological examination, cranial MRI, and consultation with a neurologist. For specific details, please refer to Supplementary Table S2.

Clinical samples, including serum and placental tissues, were collected from patients diagnosed with eclampsia and healthy controls after obtaining informed consent. The collection of these samples was approved by the institutional review board (IRB) under ethical approval number 20230006. For serum collection, blood samples were drawn from participants and centrifuged at 3,000 rpm for 10 min to separate the serum, which was stored at −80 °C until further analysis. Placental tissues were obtained during delivery, immediately placed in liquid nitrogen, and stored at −80 °C.

### 2.7 RT-qPCR

Total RNA was extracted from placental tissues and serum samples using TRIzol reagent (Thermo Fisher Scientific, Catalog No. 15596026, United States) according to the manufacturer’s instructions. cDNA synthesis was performed with 1 µg of RNA using the RevertAid First Strand cDNA Synthesis Kit (Thermo Fisher Scientific, Catalog No. K1622, United States). Quantitative PCR was carried out using SYBR Green Master Mix (Thermo Fisher Scientific, Catalog No. 4367659, United States) on an Applied Biosystems 7500 Real-Time PCR System (Thermo Fisher Scientific, United States). Primers for DPP4, IL6, TNF-α, and GAPDH were designed as follows: *DPP4* (Forward: 5′-CTC​CTG​CGG​AGG​AGA​GTC​A-3′, Reverse: 5′-GAG​CCA​TGG​AAG​GAG​GAG​A-3′); IL6 (Forward: 5′-TGA​GGA​GAC​TTG​CCT​GGT​G-3′, Reverse: 5′-AAG​GAG​GAG​ACT​TGA​GGA​AGG-3′); TNF-α (Forward: 5′-CCC​TGA​AAG​GAG​ACG​AGA​C-3′, Reverse: 5′-GAG​GAG​GAA​GGA​GGA​GAG-3′); and GAPDH (Forward: 5′-GGA​GCC​AAA​CGG​GTC​ATC​T-3′, Reverse: 5′-GAC​AGT​CAG​CCG​CAT​CTT​CT-3′).

### 2.8 Western blot

Proteins were extracted from placental tissues and serum samples using RIPA buffer (Thermo Fisher Scientific, Catalog No. 89901, United States), supplemented with protease and phosphatase inhibitors (Thermo Fisher Scientific, Catalog No. A32959, USA). Protein concentrations were determined using the BCA Protein Assay Kit (Thermo Fisher Scientific, Catalog No. 23225, United States). Equal amounts of protein (30 µg) were separated by 10% SDS-PAGE (Bio-Rad Laboratories, Catalog No. 4561033, United States) and transferred to PVDF membranes (Millipore, Catalog No. IPVH00010, United States) using a Trans-Blot Turbo Transfer System (Bio-Rad Laboratories, United States). Membranes were blocked with 5% non-fat milk (Thermo Fisher Scientific, United States) in TBST for 1 h at room temperature, followed by overnight incubation at 4 °C with primary antibodies: DPP4 (Abcam, Catalog No. ab133605, UK), IL6 (Cell Signaling Technology, Catalog No. 12153, United States), TNF-α (Cell Signaling Technology, Catalog No. 3707, USA), GAPDH (Cell Signaling Technology, Catalog No. 2118, United States), Caspase-1 (Cell Signaling Technology, Catalog No. 2267, United States), p-Caspase-1 (p17) (Cell Signaling Technology, Catalog No. 3866, United States), ASC (Santa Cruz Biotechnology, Catalog No. sc-514414, United States), p-ASC (PhosphoSolutions, Catalog No. 352-P-ASC, United States), NLRP3 (Abcam, Catalog No. ab214185, UK), p-NLRP3 (Cell Signaling Technology, Catalog No. 12291, United States), p65 (Cell Signaling Technology, Catalog No. 8242, United States), and p-p65 (Cell Signaling Technology, Catalog No. 3033, United States). After washing with TBST, membranes were incubated with HRP-conjugated secondary antibodies (Cell Signaling Technology, Catalog No. 7076, United States) for 1 h at room temperature. Protein bands were visualized using the Chemiluminescent HRP Substrate Kit (Bio-Rad Laboratories, Catalog No. 1705061, United States) and detected using a ChemiDoc Imaging System (Bio-Rad Laboratories, United States). Band intensities were quantified using ImageJ software (NIH, USA), and protein levels were normalized to GAPDH. Relative protein expression was calculated by comparing the target protein intensities between experimental and control groups.

### 2.9 Cell culture and gene knockdown with siRNA

Human Umbilical Vein Endothelial Cells (HUVECs) and the Human Trophoblast Cell Line (HTR-8) were obtained from ATCC (USA). HUVECs were cultured in Endothelial Cell Growth Medium-2 (EGM-2) (Lonza, Catalog No. CC-3162, United States), while HTR-8/SVneo cells were cultured in RPMI-1640 medium (Thermo Fisher Scientific, Catalog No. 11875093, USA), both supplemented with 10% fetal bovine serum (FBS) (Gibco, Catalog No. 16140-071, United States) and 1% penicillin-streptomycin (Thermo Fisher Scientific, Catalog No. 15140122, United States) under standard culture conditions (37 °C, 5% CO_2_). For DPP4 gene knockdown, cells were transfected with siRNA targeting *DPP4* (si-*DPP4*#1 and si-*DPP4*#2), using Lipofectamine RNAiMAX Transfection Reagent (Thermo Fisher Scientific, Catalog No. 13778075, United States). The siRNA sequences were designed as follows: si*-DPP4*#1 (5′-GGA​GGA​GAG​GAA​GAG​AAU-3′) and si-DPP4#2 (5′-GGA​AGA​GGA​GGA​AGA​GAA​U-3′), and were transfected at a final concentration of 50 nM for 48 h.

### 2.10 Transwell assay

To induce an inflammatory response, cells were treated with lipopolysaccharide (LPS) (Sigma-Aldrich, Catalog No. L2880, United States) at 1 μg/mL or TNF-α (Sigma-Aldrich, Catalog No. T0157, United States) at 20 ng/mL for 24 h. For the Transwell migration assay, Transwell inserts (Corning, Catalog No. 3422, United States) with 8 µm pores were used. Cells (1 × 10^4^) were seeded in the upper chamber in serum-free medium, and the lower chamber was filled with complete medium containing 10% FBS as a chemoattractant. After 24 h of incubation, the cells on the lower surface of the membrane were fixed with 4% paraformaldehyde (Sigma-Aldrich, Catalog No. 158127, USA) and stained with Crystal Violet (Sigma-Aldrich, Catalog No. C3886, United States). For the Transwell invasion assay, the procedure was similar, but the upper chamber of the Transwell inserts was pre-coated with Matrigel (Corning, Catalog No. 354234, United States) at 50 μg/mL for 2 h at 37 °C. After 48 h of incubation, invaded cells were fixed with 4% paraformaldehyde and stained with Crystal Violet. The number of migrated and invaded cells was quantified by counting the cells in five random fields under a light microscope (Olympus, Japan). All assays were performed in triplicate, and the data were expressed as the average number of cells per field.

### 2.11 Wound healing assay

Cells were plated in 6-well plates at a density of 5 × 10^5^ cells per well. Once cells reached confluence, a sterile 1000 µL pipette tip was used to create a uniform scratch (wound) across the center of the well. After scratching, cells were washed twice with phosphate-buffered saline (PBS) (Thermo Fisher Scientific, Catalog No. 10010023, United States) to remove any detached cells and then incubated in serum-free medium for 24 h to allow wound closure. The migration of cells into the wound area was observed and photographed at different time points (0, 12, and 24 h) under a light microscope (Olympus, Japan).

### 2.12 Tube formation assay

Growth Factor Reduced Matrigel (Corning, Catalog No. 354230, United States) was added to a 96-well plate and incubated at 37 °C for 30 min to allow gel solidification. After pre-treatment with lipopolysaccharide (LPS) (Sigma-Aldrich, Catalog No. L2880, United States) at 1 μg/mL or TNF-α (Sigma-Aldrich, Catalog No. T0157, USA) at 20 ng/mL for 24 h, HUVECs (5 × 10^4^ cells per well) were seeded onto the solidified Matrigel and incubated for 6–8 h at 37 °C in a humidified incubator with 5% CO_2_. Tube formation was observed and photographed under a light microscope (Olympus, Japan).

### 2.13 Statistical analysis

Statistical analyses were performed using R (version 4.3.1). For experimental validation, data are presented as the mean ± SEM of at least three independent biological replicates unless otherwise specified. The Student’s t-test was applied for comparisons between two groups with normally distributed data, whereas the Wilcoxon rank-sum test was used for non-normally distributed data, which are presented as medians with interquartile ranges. The specific sample size (n) for each experiment, representing the number of biologically independent samples or replicates, is stated in the corresponding figure legends. The results were considered statistically significant with the following p-value thresholds: *p* < 0.05 (*), p < 0.01 (**), and p < 0.001 (***).

## 3 Results

### 3.1 WGCNA construction and differential analysis of eclampsia transcriptome data

**FIGURE 1:**
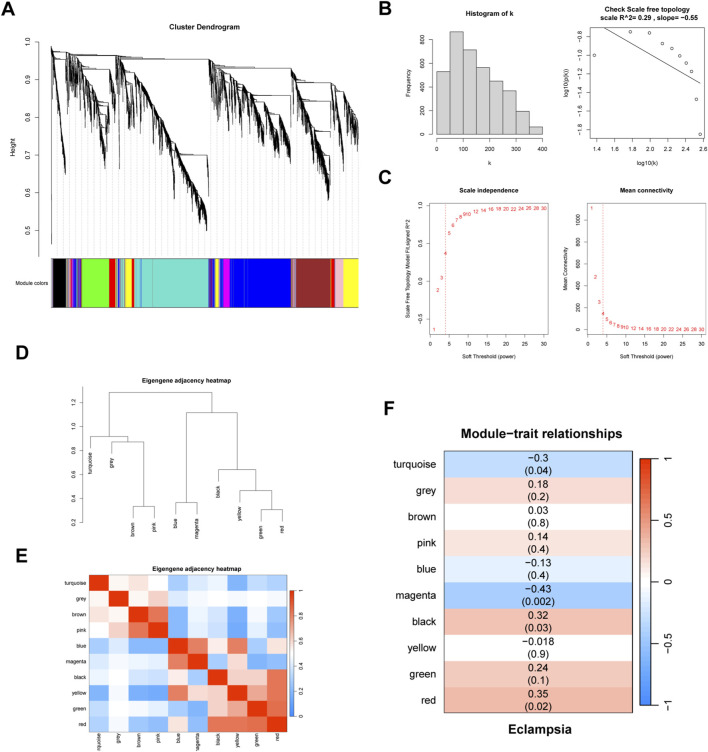
WGCNA analysis. **(A)** Cluster plot of eclampsia samples versus normal control samples. Then **(B)** scale independence and **(C)** mean connectivity. **(D)** Gene correlations were plotted as hierarchical cluster dendrograms. **(E)** HUB gene module colour markers. **(F)** Correlations between gene modules and clinical features.

To identify hub genes associated with the progression of eclampsia, we performed WGCNA. First, to explore the differential gene expression in eclampsia and its correlation with clinical traits, we generated a clustering plot comparing eclampsia samples with normal controls. The results showed clear clustering with no significant outliers (Figure 1A). A scale-free co-expression network was then constructed using a soft threshold of 4, selected based on scale independence and average connectivity (Figures 1B,C). Additionally, we created a hierarchical clustering dendrogram based on gene correlations, which revealed a total of nine gene modules (Figure 1D). These modules were color-coded for identification (Figure 1E). Finally, we assessed the correlation between the gene modules and clinical traits. The red module exhibited the highest positive correlation with eclampsia (r = 0.32, p = 0.02), while the magenta module showed the strongest negative correlation (r = −0.43, p = 0.002) (Figure 1F).

**FIGURE 2:**
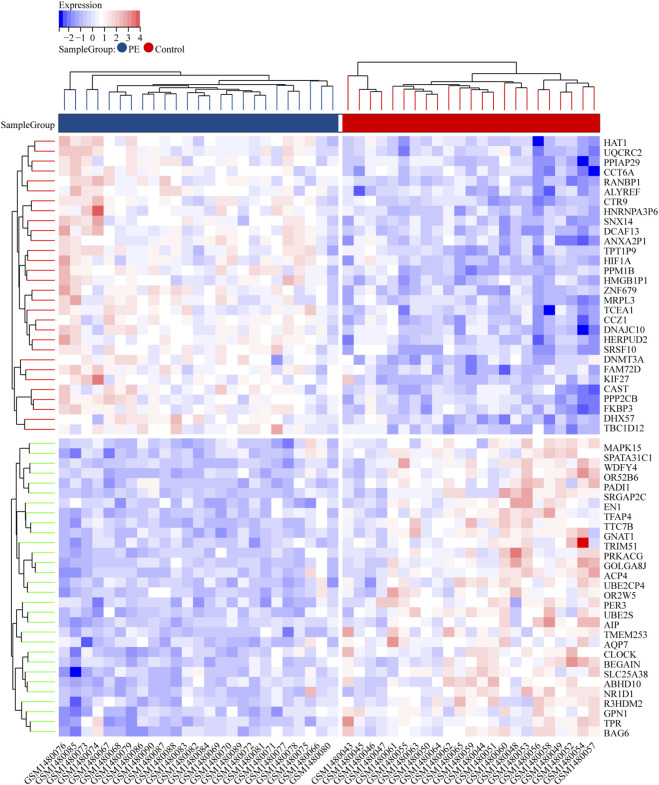
Heatmap showing a total of 4,642 upregulated genes and 2,193 downregulated genes in the eclampsia group (red) compared to the control group (blue).

To identify DEGs between the eclamptic and control groups, we analyzed the transcriptome data using R and Perl with the limma package. The thresholds for selecting DEGs were set at P < 0.05 and log |FC| > 1. For visualization, we used the ggplot2 and pheatmap packages. The heatmap analysis revealed a total of DEGs, including 4,642 upregulated genes and 2,193 downregulated genes in the eclamptic group compared to the control group (Figure 2).

### 3.2 Functional enrichment and immune cell correlation analysis of DEGs in eclampsia

**FIGURE 3:**
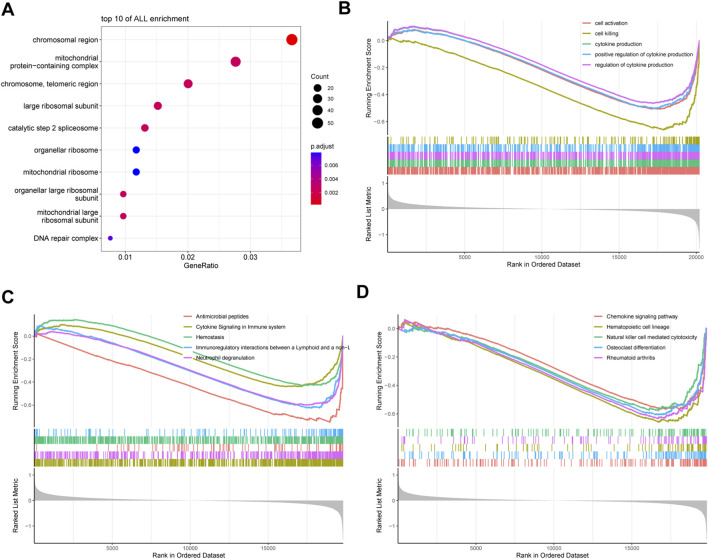
Functional enrichment analysis of eclampsia DEGs. **(A)** GO enrichment analysis. **(B)** GSEA-GO enrichment analysis. **(C)** GSEA-KEGG pathway enrichment analysis. **(D)** GSEA-Reactome pathway enrichment analysis.

To investigate the biological functions and pathways associated with DEGs in eclampsia, we performed GO and GSEA. GO enrichment analysis revealed that eclampsia DEGs were significantly associated with chromosomal regions, mitochondrial protein-containing complexes, chromosomes, telomeric regions, ribosomal large subunits, catalytic step 2 spliceosomes, organelle ribosomes, mitochondrial ribosomes, and DNA repair complexes, among other relevant processes (Figure 3A).

Further GSEA-based GO analysis identified key enrichments in processes such as cell activation, cell killing, cytokine production, and the regulation of cytokine production (Figure 3B). KEGG pathway analysis using GSEA highlighted enrichments in chemokine signaling, hematopoietic cell line development, natural killer (NK) cell-mediated cytotoxicity, osteoblast differentiation, and rheumatoid arthritis (Figure 3C). Additionally, Reactome pathway analysis identified enrichment in antimicrobial peptides, immune system cytokine signaling, hemostasis, and neutrophil degranulation (Figure 3D).

**FIGURE 4:**
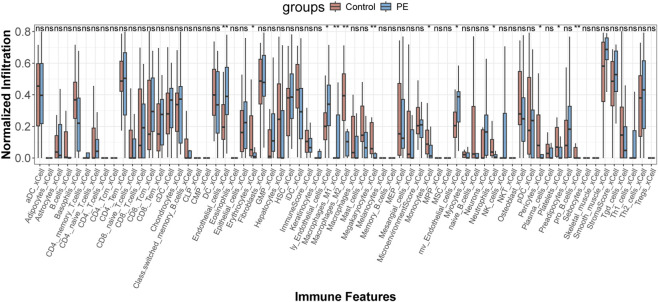
xCELL algorithm to explore how immune cells contribute to disease progression. *p* < 0.05 (*) and p < 0.01 (**).

To assess the impact of DEGs on immune cell populations in eclampsia, we used the xCELL algorithm to explore immune cell involvement in disease progression. The results revealed significant differences in the correlation of endothelial cells, erythrocytes, keratinocytes, macrophages, megakaryocytes, monocytes, neurons, and preadipocytes between the eclampsia and normal control groups (Figure 4).

### 3.3 Machine learning identification of key genes in eclampsia and expression validation

**FIGURE 5:**
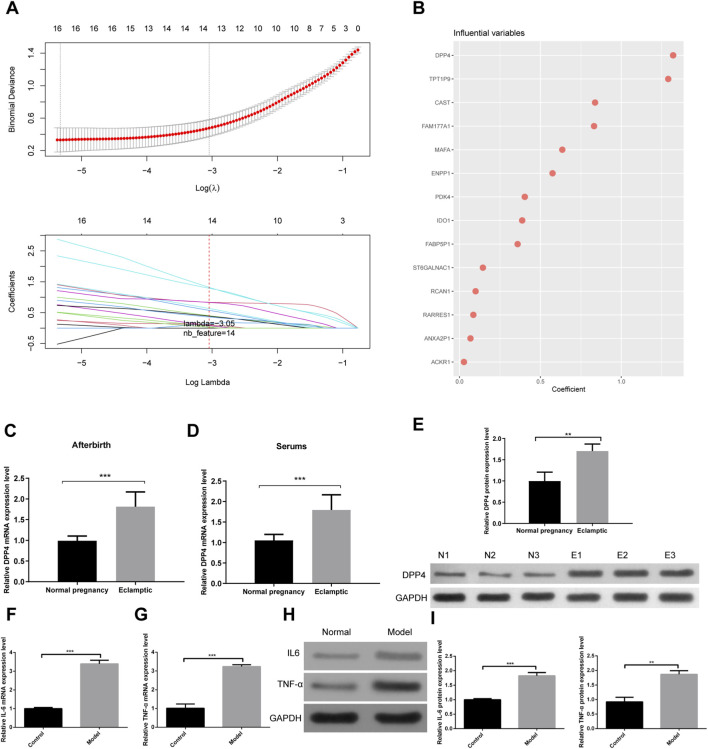
Identification and validation of DPP4 as a key regulator in eclampsia. **(A)** LASSO coefficient profile of candidate genes. The vertical dashed line indicates the optimal λ value selected by 10-fold cross-validation. **(B)** Visual representation of the 14 genes retained in the final LASSO model. **(C,D)** DPP4 mRNA expression levels in **(C)** placental tissues and **(D)** serum samples from patients with eclampsia and normotensive controls. **(E)** Representative Western blot (left) and densitometric quantification (right) of DPP4 protein in placental tissues. **(F,G)** mRNA expression levels of **(F)** IL-6 and **(G)** TNF-α in placental tissues from the study groups. **(H,I)** Protein levels of **(H)** IL-6 and **(I)** TNF-α in placental tissues, shown by representative blots (left) and their quantification (right). All quantitative data are from three independent biological replicates (n = 3) and are presented as mean ± SEM. **p < 0.01, ***p < 0.001.

To identify key genes associated with the progression of eclampsia, we performed a λ-based LASSO regression analysis (Figure 5A), which identified DPP4 as a critical gene involved in disease progression (Figure 5B). Subsequently, we collected serum and placental samples from both eclamptic patients and normal controls to assess DPP4 expression through qRT-PCR and WB assays. We found that DPP4 was significantly overexpressed in the placental tissues of eclamptic patients (Figure 5C), and a similar upregulation was observed in their serum samples (Figure 5D). Additionally, WB analysis revealed a significant increase in DPP4 protein levels in placental tissues from eclamptic patients compared to controls (Figure 5E).

Building on our previous bioinformatics analysis, which highlighted the activation of inflammatory responses in eclampsia, we further examined the expression of pro-inflammatory cytokines IL-6 and TNF-α. Both mRNA and protein levels of IL-6 and TNF-α were found to be significantly elevated in the placental tissues of eclamptic patients (Figures 5F,G). Similarly, Western blot analysis confirmed the significant upregulation of IL-6 and TNF-α proteins in the placental tissues of these patients (Figures 5H,I).

### 3.4 Construction and validation of a cellular model of eclampsia

To investigate the potential biological functions and mechanisms of DPP4 in eclampsia progression, we developed a cellular model of eclampsia-induced inflammation. Cells were stimulated with lipopolysaccharide (LPS) to induce an inflammatory response. Concurrently, we used siRNA technology to knock down DPP4 and assessed the expression levels of inflammatory cytokines IL-6 and TNF-α.

**FIGURE 6:**
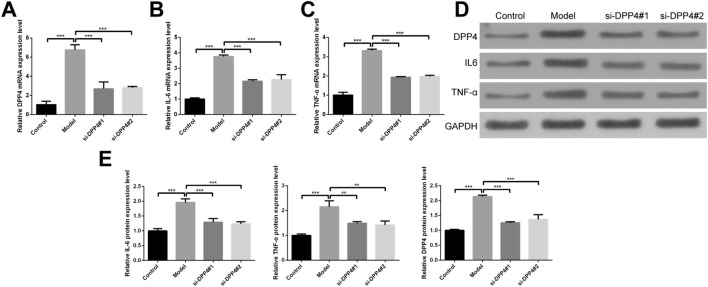
Establishment and validation of a cellular model for eclampsia-associated inflammation. **(A–C)** mRNA expression levels of **(A)** *DPP4*, **(B)** *IL-6*, and **(C)** *TNF-α* after siRNA-mediated knockdown of DPP4 in the cellular model. **(D)** Representative Western blot images showing protein expression of DPP4, IL-6, and TNF-α following DPP4 knockdown. **(E)** Densitometric quantification of protein levels from three independent biological replicates (n = 3). Data are presented as mean ± SEM. **p < 0.01, ***p < 0.001.

Our results showed that the mRNA expression of DPP4 was significantly elevated following the induction of the inflammatory response, and this increase was effectively suppressed upon DPP4 knockdown using siRNA (Figure 6A). Similarly, mRNA levels of the pro-inflammatory cytokines IL-6 and TNF-α were markedly elevated after the inflammatory response was induced, and these levels were significantly reduced upon DPP4 inhibition (Figures 6B,C).

At the protein level, DPP4 expression was also significantly upregulated following the induction of the inflammatory response, and this upregulation was reversed following DPP4 knockdown (Figure 6D). In parallel, the protein levels of IL-6 and TNF-α were significantly increased after the induction of inflammation but were substantially reduced upon DPP4 inhibition (Figure 6E). These changes were statistically significant, reinforcing the role of DPP4 in modulating the inflammatory response.

### 3.5 Migration, invasion, and lumen-forming functions of HTR-8 cells are impaired following DPP4 inhibition

Following successful inhibition of DPP4 in HTR-8 cells, we evaluated the impact on cell biological functions using Transwell migration, invasion, scratch, and lumen formation assays.

**FIGURE 7:**
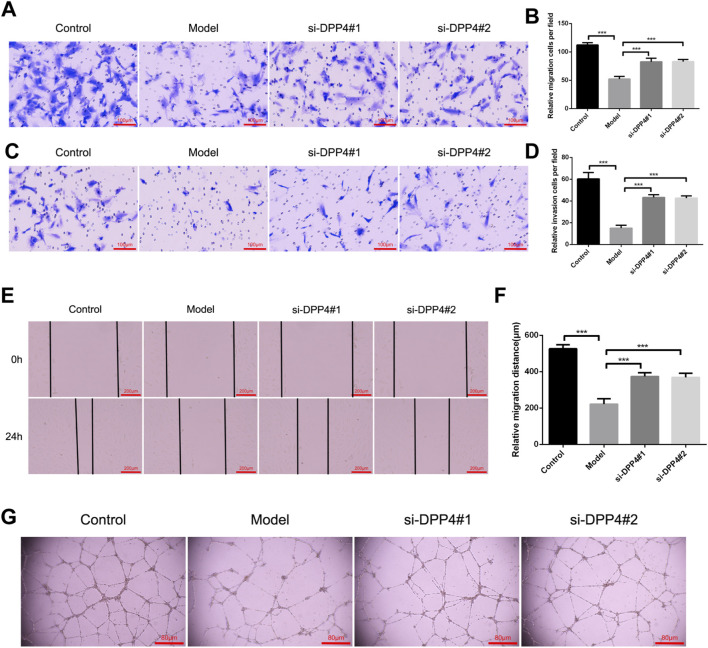
Functional consequences of DPP4 inhibition on HTR-8 trophoblast cells. **(A)** Representative images of Transwell migration assays (scale bar: 100 µm) and **(B)** corresponding quantification. **(C)** Representative images of Matrigel-based Transwell invasion assays (scale bar: 100 µm) and **(D)** corresponding quantification. **(E)** Representative images of scratch-wound healing assays at 0 h and 24 h (scale bar: 200 µm) and **(F)** quantification of wound closure. **(G)** Representative images and quantification of tube-formation assays in Matrigel. All quantitative data are derived from three independent biological replicates (n = 3) and are presented as mean ± SEM. ***p < 0.001.

In the Transwell migration assay, we observed a significant reduction in the migratory capacity of HTR-8 cells upon DPP4 inhibition, compared to the control group (Figures 7A,B). Similarly, in the Transwell invasion assay, DPP4 inhibition led to a marked decrease in the invasion ability of HTR-8 cells (Figures 7C,D). In addition, a scratch assay further confirmed that DPP4 inhibition significantly reduced the migratory potential of HTR-8 cells at both 0 and 24 h, relative to the control group (Figures 7E,F). Finally, we assessed the lumen-forming ability of HTR-8 cells through a lumen formation assay. DPP4 inhibition resulted in a significant restoration of the cells’ lumen-forming capacity compared to the control group (Figure 7G).

### 3.6 Inhibition of the p65/NLRP3/ASC/Caspase-1 pathway phosphorylation after DPP4 depletion

**FIGURE 8:**
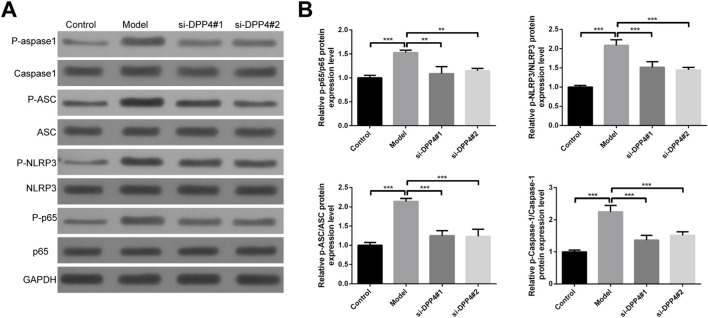
Western blot analysis of protein expression and phosphorylation in the p65/NLRP3/ASC/Caspase-1 signaling pathway following DPP4 inhibition. **(A)** Representative immunoblots showing protein and phosphorylation levels of p65, NLRP3, ASC and caspase-1 after DPP4 knockdown or inhibition. **(B)** Quantitative analysis of band intensity from three independent biological replicates (n = 3). Data are presented as mean ± SEM. **p < 0.01, ***p < 0.001.

To investigate the mechanisms underlying the effects of DPP4 on biological functions in an inflammatory response model of HTR-8 cells, we examined the protein expression and phosphorylation levels of key components in the p65/NLRP3/ASC/Caspase-1 signaling pathway. Our results demonstrated that phosphorylation levels of p65, NLRP3, ASC, and Caspase-1 were significantly reduced following DPP4 depletion, compared to the model group (Figure 8A). Statistical analysis of grey-scale data further confirmed that these changes were significant (Figure 8B).

## 4 Discussion

Eclampsia exerts a profound influence on maternal and neonatal health, accounting for a considerable fraction of emergency department presentations, maternal fatalities, and enduring complications (Sibai and Stella, 2009). The ascertainment of dependable biomarkers is imperative for augmenting the diagnosis, prophylaxis, and administration of eclampsia (Guglielminotti et al., 2023; MacDonald et al., 2022). Additionally, probing into the aberrant inflammatory response and other etiological mechanisms is pivotal for devising more efficacious early predictive markers and therapeutic stratagems. In our investigation, we harnessed transcriptomic data derived from eclampsia cases to scrutinize the potential pathogenic mechanisms underlying eclampsia progression via differential expression analysis and functional enrichment studies. Subsequently, employing WGCNA in conjunction with machine learning methodologies, we pinpointed the key gene DPP4 implicated in eclampsia progression. Ultimately, we corroborated the biological role and potential mechanism of DPP4 in eclampsia progression through clinical specimen analysis and cellular experimental validation.

Our investigation has illuminated novel aspects of the pathophysiological mechanisms underpinning eclampsia progression, achieved through the identification of key DEGs utilizing WGCNA, differential expression analysis, and functional enrichment studies. These analyses have unveiled significant correlations with cellular processes and immune responses. Specifically, our findings suggest that the etiology of eclampsia is multifactorial, encompassing an imbalance of angiogenic factors, immune dysregulation, and placental dysfunction. Notably, placental-derived galectin-9 has emerged as a novel biomarker for the early prediction of eclampsia in early pregnancy maternal blood (Li et al., 2018; Mittelberger et al., 2022). This biomarker is implicated in the promotion of preeclampsia development by inducing defects in uterine vascular remodeling, a process mediated through the modulation of pro-inflammatory macrophage subpopulations.

Our investigation employed LASSO regression, a machine learning technique, to identify key genes implicated in the progression of eclampsia, pinpointing DPP4 (Dipeptidyl peptidase-4) as a potential pivotal gene. DPP4, a ubiquitously expressed protease, is primarily engaged in protein degradation and plays a crucial role in immune responses, glucose metabolism, and inflammatory processes (Barchetta et al., 2022). Research has demonstrated that DPP4 overexpression is linked to various chronic conditions and acute pathologies, including diabetes mellitus, cardiovascular diseases, and certain immune-related disorders (Nargis and Chakrabarti, 2018; Maanvi and Deshmukh, 2023; Duan et al., 2017). To date, no studies have directly correlated DPP4 overexpression with eclampsia development. Furthermore, the pro-inflammatory cytokines IL-6 and TNF-α were found to be significantly upregulated in the placental tissues of patients with eclampsia, reinforcing the association between eclampsia and systemic inflammatory responses (Abdelzaher et al., 2022). This suggests an aberrant activation of the immune system in affected individuals, leading to an overproduction of pro-inflammatory cytokines by immune cells. These cytokines may exacerbate hypertension and multi-organ dysfunction during pregnancy through mechanisms such as vascular endothelial cell injury, increased vascular permeability, and coagulation system activation. Concurrently, the placenta serves as a critical exchange interface between the mother and fetus. Elevated levels of inflammatory factors like IL-6 and TNF-α can alter the placental microenvironment, thereby impacting oxygen and nutrient delivery, potentially resulting in fetal growth restriction or intrauterine hypoxia.

Recent studies have demonstrated that DPP4 is not merely an enzyme but also plays a significant role in immune regulation, modulating the immune response by influencing cytokine profiles and leukocyte activity. In this study, we employed HTR-8 cells, a placental trophoblast cell line, to investigate the impact of DPP4 inhibition on cellular functions. Knockdown of DPP4 resulted in a marked reduction in cell migration, invasion, and lumen formation, all of which are essential for normal placental development. Trophoblast cells must invade the uterine wall and form structures that support fetal development, and this process is critical for a successful pregnancy. Impaired trophoblast invasion and abnormal placental development are closely linked to preeclampsia and eclampsia. Following DPP4 inhibition, the migration and invasion of HTR-8 cells were significantly diminished, and lumen formation was substantially impaired, reflecting the pathophysiological mechanisms underlying eclampsia. In the context of eclampsia, shallow placental invasion results in poor placental perfusion, exacerbating maternal hypertension. Thus, DPP4 inhibition may improve placental function and mitigate this pathological process. In conclusion, DPP4 appears to promote excessive inflammatory responses that contribute to vascular injury and placental dysfunction in eclampsia. Targeting DPP4 may represent a promising therapeutic strategy to modulate the inflammatory response, alleviate maternal symptoms, and prevent associated complications.

The p65/NLRP3/ASC/Caspase-1 axis is a critical component of the inflammasome pathway, playing a pivotal role in regulating the inflammatory response by facilitating the activation of pro-inflammatory cytokines such as IL-1β and IL-18 (Ye et al., 2024). In our study, we observed a significant reduction in the phosphorylation levels of p65, NLRP3, ASC, and Caspase-1 following DPP4 inhibition, suggesting that DPP4 may be integral to modulating the inflammatory response in trophoblast cells. p65, a subunit of NF-κB, is phosphorylated as a key step in the activation of the NF-κB signaling pathway, which serves a central role in the inflammatory cascade (Fuentes et al., 2016). Likewise, NLRP3, ASC, and Caspase-1 are essential components of the NLRP3 inflammasome, and their activation leads to the cleavage of pro-Caspase-1 into its active form (Kelley et al., 2019; Shao et al., 2015). Activated Caspase-1 further processes pro-inflammatory cytokines, such as IL-1β, converting them to their active forms and thereby amplifying the inflammatory response.

While our integrated bioinformatic and experimental approach identifies DPP4 as a novel regulator of the p65/NLRP3/ASC/Caspase-1 pathway in eclampsia, several limitations should be noted. The clinical validation cohort, while rigorously characterized, had a modest sample size and specifically compared eclampsia against normotensive controls; a dedicated preeclampsia group was not included to definitively distinguish whether DPP4 upregulation is a general marker of placental stress or a specific driver of progression to eclampsia. Furthermore, although we observed elevated serum DPP4, our study focused on its local placental mechanism, and the potential role of soluble DPP4 as a systemic inflammatory mediator affecting distant organs remains a compelling hypothesis for future investigation.

The observed reduction in the phosphorylation of these key inflammasome components after DPP4 depletion is noteworthy. First, it suggests that targeting DPP4 could provide a promising therapeutic approach to control the inflammatory response in eclampsia. Inhibiting this pathway could potentially reduce the production of pro-inflammatory cytokines, thereby alleviating systemic inflammation that exacerbates maternal symptoms. Furthermore, inhibition of this inflammatory signaling pathway may restore trophoblast cell functions such as migration, invasion, and lumen formation, which are essential for normal placental development and function.

Looking forward, our findings directly highlight a translational opportunity. Given that DPP4 inhibitors (gliptins) are already clinically available with established safety profiles for other conditions, drug repurposing represents a logical and promising next step (Grouzmann et al., 2007; Author anonymous, 2016). Future preclinical studies in relevant animal models are warranted to evaluate the efficacy and safety of gliptins in mitigating eclampsia-associated pathology. Subsequently, carefully designed clinical trials could assess their potential to prevent or treat severe hypertensive disorders of pregnancy. Additionally, expanding validation to larger, stratified cohorts that include PE patients will be crucial to determine the specificity of DPP4 as a biomarker for disease progression.

In conclusion, our findings offer compelling evidence that DPP4 contributes to the disruption of normal placental function by promoting the activation of the p65/NLRP3/ASC/Caspase-1 inflammasome pathway, thereby fostering an inflammatory environment. Targeting DPP4 may represent an innovative strategy to modulate inflammatory signaling in eclampsia, with the potential to mitigate maternal symptoms and improve pregnancy outcomes.

## 5 Conclusion

Our study highlights DPP4 as a crucial mediator in eclampsia progression, where its inhibition reduces inflammatory responses and restores trophoblast functions critical for placental development, offering a promising therapeutic strategy to alleviate maternal symptoms and improve pregnancy outcomes.

## Data Availability

The original contributions presented in the study are publicly available. This data can be found in the GEO data repository with the accession number GSE60438.
