# Supplementary material for: Integrative biology shows DPP4 affects inflammatory response to eclampsia and cell model growth via p65/NLRP3/ASC/Caspase-1 pathway
Source: Front Genet. 2026 Mar 4;17:1775026. doi: 10.3389/fgene.2026.1775026 (PMC12995188; doi:10.3389/fgene.2026.1775026)
Supplement: Supplementary file 1 [file Table1.docx]

****Supplementary Table 1. Baseline Clinical and Demographic Characteristics of the Study Cohort.****

| **Characteristic** | **Normotensive Control**  **(n=25)** | **Preeclampsia**  **(n=23)** | **p-value** |
| --- | --- | --- | --- |
| ****Maternal Age (years)****a | 32.3 ± 0.5 | 31.2 ± 0.8 | 0.24 |
| ****Gestational Age at Delivery (weeks)****a | 39.1 ± 0.1 | 32.3 ± 0.5 | ****<0.001**** |
| ****Gravidity**** |  |  | ****<0.001**** |
| Primigravida | 29 (44.6%) | 40 (66.7%) |  |
| Multigravida | 36 (55.4%) | 20 (33.3%) |  |
| ****Parity**** |  |  | ****<0.001**** |
| Nulliparous | 32 (49.2%) | 51 (85.0%) |  |
| Parous | 33 (50.8%) | 9 (15.0%) |  |
| ****Systolic BP (mmHg)****a | <140 | 172.2 ± 1.9 | ****<0.001****b |
| ****Diastolic BP (mmHg)****a | <90 | 105.6 ± 1.1 | ****<0.001****b |
| ****Antihypertensive Treatment**** | 0 (0%) | 48 (80.0%) | ****<0.001**** |
| ****MgSO₄ Treatment**** | 0 (0%) | 37 (61.7%) | ****<0.001**** |
| ****Infant Sex**** |  |  | 0.48 |
| Female | 33 (50.8%) | 27 (45.0%) |  |
| Male | 32 (49.2%) | 34 (55.0%)c |  |
| ****Infant Birth Weight (g)****a | 3369.2 ± 54.4 | 1738.1 ± 125.3 | ****<0.001**** |
| ****Infant Weight Percentile****d | 25-50 | 10-25 | ****<0.001**** |

****Notes:****
a Data presented as mean ± SEM.
b p-value for blood pressure comparisons derived from one-sample t-test against the diagnostic threshold (140/90 mmHg).
c Includes one twin pregnancy in the preeclampsia group.
d Data presented as median range.
BP: Blood pressure; MgSO₄: Magnesium sulfate.
Statistical tests: Student's t-test with Welch's correction for continuous data; Fisher's Exact Test for categorical data.
