# Supplementary material for: Integrative biology shows DPP4 affects inflammatory response to eclampsia and cell model growth via p65/NLRP3/ASC/Caspase-1 pathway
Source: Front Genet. 2026 Mar 4;17:1775026. doi: 10.3389/fgene.2026.1775026 (PMC12995188; doi:10.3389/fgene.2026.1775026)
Supplement: Supplementary file 2 [file Table2.docx]

| **Characteristic** | **Normotensive Control (n=3)** | **Eclampsia (n=3)** |
| --- | --- | --- |
| ****Maternal Age (years)**** | 32.0 ± 1.0 | 31.7 ± 1.5 |
| ****Gestational Age at Delivery (weeks)**** | 39.3 ± 0.6 | 32.0 ± 0.0 |
| ****Systolic BP (mmHg)**** | 118.3 ± 3.2 | 174.7 ± 5.5 |
| ****Diastolic BP (mmHg)**** | 75.7 ± 2.9 | 106.3 ± 2.3 |
| ****Proteinuria (g/24h)**** | Not detected | 4.8 ± 0.6 |
| ****Neonatal Birth Weight (g)**** | 3416.7 ± 102.1 | 1683.3 ± 91.3 |

**Supplementary Table S2**

Data are presented as mean ± SEM. BP, blood pressure.
All sample collection procedures were approved by the relevant Institutional Review Board, and informed consent was obtained from all participants.
